# On the Treatment of Phthisis Pulmonalis

**Published:** 1848

**Authors:** Hughe Bennett


### ARTICLE VII.

*On the treatment of Phthisis Pulmonalis by Cod Liver Oil.* By DR. HUGHE BENNETT.

The effect of the oil in many cases of phthisis, is very striking, and is well seen in hospital and dispensary practice. Individuals presenting emaciation, profuse sweats, constant cough and expectoration, as most prominent symptoms, with a degree of weakness that prevents their standing alone, after a few weeks' use of it are enabled to get up with ease and walk about, with a visible improvement in their general health, and an increased amount of flesh. The physical signs of the disease may continue unaffected for some time; but if the treatment be continued, the moist gurgling rales are exchanged for dry blowing sonnds, which become more and more persistent, pectoriloquy is merged into bronchophony, the respiration is easier, and a check is evidently given to the ulcerative process, and the formation of purulent matter in the air passages. In this state, patients often feel themselves so well, that they insist on leaving the hospital, or give up their attendance on the dispensary. Dr. Bennett has frequently found it impossible to prevail on such persons to continue the treatment, and the consequence is, that, again returning to their often unhealthy employment and bad diet, and exposed to the other causes favorable to the production of the disease, the distressing symptoms again recur. Several cases, with one or more caverns in the lungs, have, in this manner, returned to the Infirmary from four to seven or eight times during the last six years, and on each occasion have gone out, in their own opinion, perfectly cured.

Notwithstanding the difficulties which have presented themselves in bringing about a complete cure of the disease, Dr. Bennet has succeeded, in several cases, in ascertaining that caverns have completely healed up, every symptom and physical sign indicating their presence, having disappeared, and only slight dulness on percussion, and increased vocal resonance remaining as a proof of the puckering and induration of the pulmonary parenchyma attendant on the cicatrix. He gives two unequivocal cases where it occurred, and alludes to others which he proposes publishing at some future time.

Most cases of phthisis pulmonalis, especially in the advanced stage, are affected with more or less dyspepsia, which renders the stomach irritable, causes total loss of appetite, and is often the cause that prevents nourishment from being taken. In many instances there is no difficulty in employing the oil under these circumstances, but in others it cannot be retained on the stomach. It will then be necessary to calm the irritability of the organ, and the best remedy for this purpose, according to Dr. B.'s experience, is naphtha. It is to the power this substance has of checking vomiting, and thereby allowing nourishment to be retained, that he attributes the advantages which have attended its use in the practice of Dr. J. Hastings, and others. The diet should always be nutritive, without being stimulating; and counter-irritation to the chest, is an excellent auxiliary. This treatment should be perseveringly persisted in; whilst, to prevent fresh exudations of tubercular matter, an equable temperature is of the highest importance. To equable temperature must be ascribed the advantages of favored localities for phthisis, and with proper precautions it can be very well maintained in this climate.—*Monthly Journal Med. Sciences, in Medical Examiner.*

